# Role of Full Outline of Unresponsiveness (FOUR) Score and Glasgow Coma Scale (GCS) in critically ill children to predict outcome: A prospective observational study

**DOI:** 10.1590/1984-0462/2026/44/2025181

**Published:** 2026-07-17

**Authors:** Bageshree Saha, Sanjay Halder, Moumita Samanta, Saikat Mahato, Sandipan Sen, Tapan Kumar Sinhamahapatra

**Affiliations:** a Nil Ratan Sircar Medical College and Hospital, Kolkata, West Bengal, India.; b Medical College and Hospital, Kolkata, West Bengal, India.

**Keywords:** Prognosis, Pediatric Intensive Care Units, Hospital mortality, Coma, Prognóstico, Unidades de Terapia Intensiva Pediátrica, Mortalidade hospitalar, Coma

## Abstract

**Objective:**

To evaluate the diagnostic value of the Outline of Unresponsiveness (FOUR) Score and Glasgow Coma Scale (GCS) to predict mortality in critically ill children with non-traumatic impaired consciousness.

**Methods:**

In this single-center prospective observational study, children aged one month to 12 years admitted to the pediatric Intensive Care Unit (PICU) were included, and serial FOUR score and GCS at admission, 12^th^, and 24^th^ hour were recorded. Children with PICU stay of <24 hours, with convulsion within the last hour, visual or motor impairment, or use of sedatives/ neuromuscular relaxants were excluded. Primary outcome: in-hospital mortality. Secondary outcome: requirement of intubation, and comparison of two scores for mortality.

**Results:**

One hundred critically ill children were enrolled with a median (interquartile — IQR) age of 46.0 (10.0–84.0) months, and 53 children died during the hospital course. The GCS and FOUR scores were significantly higher among survivors (p<0.001). Both scores were independent predictors of mortality at admission and the 12^th^ hour, but only the FOUR score was a statistically significant predictor of mortality at the 24^th^ hour. The adjusted odds ratio (AOR) for mortality for FOUR24 was 0.505 (95% confidence interval — 95%CI 0.399–0.863) and for GCS24 score was 2.728 (95%CI 0.932–2.273). Sixty-three children needed intubation, with a median duration of 4.0 (1.0–7.0) days, and of these, 69.8% died.

**Conclusions:**

GCS and FOUR scores have comparable efficacy in predicting mortality during initial assessment. However, the FOUR score is better than GCS at the 24^th^ hour and can hence be considered a prognostic tool.

## RESUMO

**Objetivo:**

Avaliar o valor diagnóstico do escore Full Outline of Unresponsiveness (FOUR) e da Escala de Coma de Glasgow (GCS) para predizer a mortalidade em crianças gravemente doentes, com comprometimento não traumático da consciência.

**Métodos:**

Estudo observacional prospectivo, unicêntrico, que inclui crianças de um mês a 12 anos internadas na Unidade de Terapia Intensiva Pediátrica (UTIP). Os escores FOUR e GCS foram registrados de forma seriada na admissão, na 12a e na 24a hora. Foram excluídas crianças com permanência na UTIP <24 horas, convulsão na última hora, deficiência visual ou motora ou uso de sedativos/relaxantes neuromusculares.

**Resultados:**

Cem crianças gravemente doentes foram incluídas, com idade mediana (intervalo interquartil — IIQ) de 46,0 (10,0–84,0) meses, e 53 evoluíram a óbito durante a internação. As médias da GCS e do escore FOUR foram significativamente maiores entre os sobreviventes (p<0,001). Ambos os escores foram preditores independentes de mortalidade na admissão e na 12a hora; apenas o escore FOUR foi preditor estatisticamente significativo na 24a hora. A razão de chances ajustada (AOR) para mortalidade foi de 0,505 (intervalo de confiança de 95% — IC95% 0,399–0,863) para FOUR24 e de 2,728 (IC 0,932–2,273) para GCS24. Sessenta e três crianças necessitaram de intubação, com duração mediana de 4,0 (1,0–7,0) dias, das quais 69,8% evoluíram a óbito.

**Conclusões:**

Os escores GCS e FOUR apresentam eficácia comparável na previsão de mortalidade na avaliação inicial. Entretanto, o escore FOUR apresentou melhor desempenho na 24a hora e pode, portanto, ser considerado uma ferramenta de prognóstico.

## INTRODUCTION

 Neurological evaluation in children with altered sensorium is challenging and crucial in emergency and critical care settings. The pediatric Glasgow Coma Scale (pGCS) is widely adopted for evaluating unconscious children. Although historically designed for head trauma assessment,^1^ the GCS was later validated for non-traumatic coma^2^ as a therapeutic indicator of disease severity and outcomes.^3,4^ However, limitations of the GCS include poor interobserver agreement, limited utility in intubated patients, and variable predictive value.^5^ To address these limitations, a novel coma scale, "Full Outline of Unresponsiveness (FOUR) score," has been proposed by excluding the verbal component and incorporating brainstem reflexes and respiration. Although found useful in adults, its validation in children with altered sensorium remains limited.^5-8^ Previous studies report an area under the receiver-operating characteristic curve (AUC-ROC) of 0.77–0.96 for GCS in predicting pediatric mortality, whereas pediatric data for the FOUR score are scarce. The largest Indian pediatric study reported an AUC-ROC of 0.940 for mortality prediction using the FOUR score, indicating promising performance.^9^ However, further evidence is required to validate its use in children in Intensive Care Unit (ICU) settings, particularly in non-traumatic coma. 

 In this study, we evaluated the utility of the FOUR score and pGCS in predicting outcomes among children admitted to the pediatric intensive care unit (PICU) with non-traumatic impaired consciousness. We also compared the predictive accuracy of both scores for in-hospital mortality. 

## METHOD

**Figure 1:**
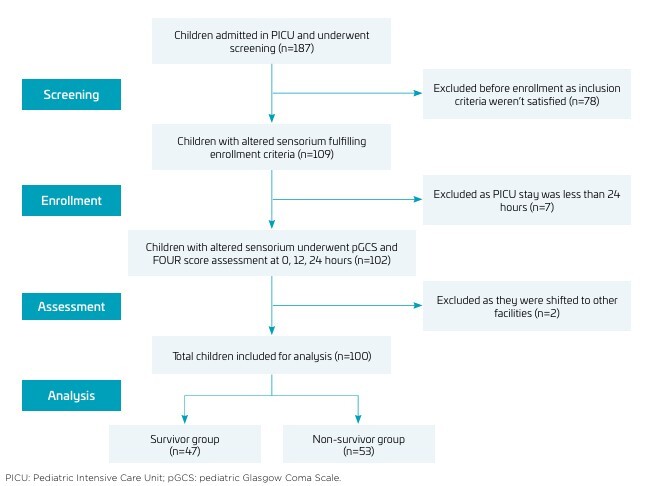
Study flowchart as per Strengthening the Reporting of Observational Studies in Epidemiology guidelines.

 This observational study was carried out in the PICU of a tertiary care center in Eastern India. It was designed, conducted, and reported in accordance with the Strengthening the Reporting of Observational Studies in Epidemiology (STROBE) statement (Figure 1). Children aged one month to 12 years, admitted to the PICU, were included. The study lasted two years, from June 1, 2021, to May 31, 2023. 

 Ethical clearance was obtained from the institutional review committee in advance. Purposive sampling was used for subject recruitment after obtaining written informed consent from the respective parents/guardians. The protocol followed the principles of the Declaration of Helsinki. 

 The study enrolled children admitted to a non-trauma PICU with altered sensorium and a minimum ICU stay of 24 hours. Exclusion criteria were seizure within the previous one hour, visual or motor impairment, use of sedatives or neuromuscular blockers within the last two hours, and mechanical ventilation at admission. For sample size calculation, based on previous reports, the AUC-ROC values for the GCS and FOUR scores are 0.865 and 0.945, respectively.^9^ The correlation between scores and the outcome was set at 0.7, with α=0.05 and β=0.20. The calculated sample size was 101, using PS: Power and Sample Size Calculation (Version 3.1.6, Department of Biostatistics, Vanderbilt University School of Medicine, Nashville, USA). 

 The pGCS consists of three heterogeneous domains of eye, verbal, and motor responses; a lower score represents more limitation in responses.^10^ The modified FOUR score, validated in infants and children by Czaikowski et al,^11^ was also applied. It contains four components of eye response, motor response, brainstem reflexes, and respiration, each graded from 0 to 4. All the raters were senior resident doctors posted in the PICU. They received training in FOUR scores during a 2-week workshop led by a neurologist and an intensivist. Their hands-on performances were overseen by the PICU in charge for the following two weeks. A four-step approach of check, observe, stimulate, and rate was used for serial assessment. The rating is performed against defined criteria in a standard, structured sequence: first, whether the patient’s findings meet the criterion for the top step for each mode of behavior measured in the GCS is assessed. If a criterion is satisfied, the corresponding rating is assigned; if not, subsequent steps are considered in a descending sequence until we confirm there’s no response. If the assessed component could not be assessed, it was marked as "not testable" (NT), especially in intubated patients, where verbal (V) was rated as NT. However, for analysis, all V (NT) values were set to V1 to obtain a total GCS score. Similarly, the FOUR score was applied. Patient management decisions were taken independently by the treating PICU team. Scores were recorded at admission, 12 hours, and 24 hours. 

 Data were recorded methodically in a predesigned structured form containing detailed history, clinical examination, and serial pGCS and FOUR scores, details of treatment, imaging, and laboratory investigations. Variables extracted for analysis included age, sex, diagnosis, physiological derangement (shock, respiratory distress, coma, and status epilepticus), organ dysfunction (cardiovascular, respiratory, neurologic, hematologic, renal, and hepatic) at admission, requirement of inotropes, and respiratory support (invasive and non-invasive ventilation). Severity of multi-organ involvement was assessed using the pediatric sequential organ function assessment (pSOFA) score.^12^ Hemodynamic support was evaluated using the highest vasoactive-inotropic score (VIS), which is calculated by summing the doses of different inotropes and vasopressors.^13^

 The children were further followed up during their hospital course. The primary outcome was in-hospital mortality (discharge versus death). The secondary outcome of the study was the need for endotracheal intubation, and comparison of AUC-ROC values of pGCS and FOUR scores for mortality prediction. ICU-free days were calculated as 30 minus ICU stay duration; deaths were considered as zero (0). 

 Data were entered in a tabular format in a Microsoft Excel spreadsheet and analyzed using Statistical Package for the Social Sciences — SPSS (version 26). Normality was examined using the Kolmogorov-Smirnov test. Normally distributed variables were represented using mean±standard deviation (SD), and median (interquartile range — IQR) for nonparametric variables. Between-group differences were evaluated using independent-samples t-test or Mann-Whitney U test. Categorical variables were compared using Pearson’s chi-square and Fisher’s exact test. A p<5% was statistically significant. 

 ROC curves were plotted to compare the predictive values of pGCS and FOUR for mortality. AUC-ROCs were calculated and compared for GCS (GCS-0, GCS-12, GCS-24) and FOUR (FOUR-0, FOUR-12, FOUR-24) scores. Optimal cut-offs for pGCS and FOUR scores were determined using the Youden index. Their sensitivity, specificity, positive predictive value (PPV), and negative predictive value (NPV) were calculated. The association between subsequent pGCS and FOUR scores and in-hospital mortality was evaluated using logistic regression. Multiple logistic regression models using the enter method were performed to analyze the association between the subsequent scores (both GCS and FOUR) and mortality. The Hosmer-Lemeshow test and Nagelkerke R-squared were used to assess whether the models fit the data. 

## RESULTS

 During the study period, 109 children with altered sensorium were admitted to the PICU. Seven children died within 24 hours and were excluded; two were transferred to other facilities (Figure 1). Of the 100 children evaluated, 53 were males (male:female ratio 1.1:1). The median (IQR) age was 46.0 (10.0–84.0) months. Forty-eight children had primary neurological abnormalities, while 52 had secondary neurological manifestations. Neuroinfection (52%) was the commonest cause. Secondary infective encephalopathy includes children with altered sensorium having a primary infective focus other than the Central Nervous System (CNS), which includes dengue encephalopathy (6), scrub encephalitis (4), severe malaria (2), and septic shock without primary CNS infection (8). Metabolic disorders (22 children), followed by intracranial space-occupying lesions (SOL), were the most frequent non-infectious cause. Metabolic causes included diabetic ketoacidosis (7), hepatic encephalopathy (4), neurotoxic snakebite (3), organophosphorus poisoning (2), and chronic kidney disease (CKD) with uremic encephalopathy (6). The clinical-demographic variables of the study population are shown in Table 1. The median interval from symptom onset to PICU admission was 4.0 (2.0–6.0) days, comparable between both groups. 

**Table 1:** Clinical-demographic characteristics of the critically ill children with altered sensorium, admitted in the pediatric Intensive Care Unit (n=100):

		Total population	Survivors (n=47)	Non-survivors (n=53)	p-value
Ages (months)		46.0 (10.0–84.0)	30.0 (8.0–84.0)	53.0 (11.0–84.0)	
	≤12 months	28 (28.0)	14 (29.7)	14 (26.4)	0.516
	1–5 years	32 (32.0)	16 (34.1)	16 (30.2)	
	>5 years	40 (40.0)	17 (36.2)	23 (43.4)	
Gender, Male		53 (53.0)	25 (53.2)	28 (52.8)	0.565
Diagnosis					
Infectious causes					
	AES	25 (25.0)	10 (21.3)	15 (28.3)	
	Secondary encephalopathy*	20 (20.0)	16 (34.1)	4 (7.6)	
	Meningitis	7 (7.0)	5 (10.5)	2 (3.8)	
Non-infectious causes					
	Metabolic disorders†	22 (22.0)	9 (19.1)	13 (24.5)	
	ICH/SOL	15 (15.0)	1 (2.1)	14 (26.4)	
	Acute stroke syndrome	4 (4.0)	2 (4.2)	2 (3.8)	
	Post-operation	3 (3.0)	1 (2.1)	2 (5.7)	
	Electrolyte abnormalities	2 (2.0)	2 (4.2)	0	
	Epileptic disorders	2 (2.0)	1 (2.1)	1 (1.9)	
Anthropometric indicators					
	Weight for height (Z)	-0.63 (-3.1;0.3)	-0.46 (-2.2;0.41)	-1.56 (-3.5;0.2)	0.178
	Height for age (Z score)	-0.58(-1.8;0.3)	-0.57 (-1.7;0.2)	-0.89 (-1.9;0.6)	0.928
	BMI for age (Z score)	-1.02 (-3.3;0.8)	-0.44 (-2.9;1.2)	-1.29 (-3.6;0.7)	0.091
	PICU window (days)‡	4.0 (2.0;6.0)	4.0 (2.0;5.0)	4.0 (1.5;7.0)	0.464
	PICU stay (days)	7.0 (3.0;14.0)	12.0 (4.0;15.0)	5.0 (2.0;9.5)	<0.001
	pSOFA on admission	9.0 (4.0)	7.0 (5.5)	13.0 (4.0)	<0.001
	Intubated	63 (63)	19 (17.0)	44 (83.0)	<0.001
	IMV Duration (days)	4.0 (1.0-7.0)	5.0 (1.5-14.0)	3.0 (1.0-7.0)	0.249
Inotropes requirement					
	No needed	20 (20.0)	20 (42.6)	0	
	≤2 inotropes	29 (29.0)	20 (42.6)	9 (16.9)	
	>2 inotropes	51 (51.0)	7 (14.9)	44 (83.0)	

*This group includes secondary encephalopathy with a primary infective focus other than CNS. Among 20 children with secondary infective encephalopathy, 6 children had dengue encephalopathy, 4 had scrub encephalitis, 2 had severe malaria, and the remaining 8 children developed septic shock without any primary CNS infection; †Seven children with diabetic ketoacidosis (DKA), four with hepatic encephalopathy, 3 children suffering neurotoxic snakebite, 2 organophosphorus poisoning, and six having chronic kidney disease (CKD) with uremic encephalopathy were included in this metabolic encephalopathy group; ‡PICU window refers to the interval (in days) between onset of first symptom and PICU admission.

AES: Acute encephalitic syndrome; ICH: Intracerebral hemorrhage; SOL: Space-occupying lesion; BMI: Body mass index; pSOFA: Pediatric sequential organ failure assessment; IMV: Invasive mechanical ventilation; PICU: Pediatric Intensive Care Unit.

 Among the studied children, 47 survived and were discharged from the PICU. Meningoencephalitis/ acute encephalitis syndrome (AES) had the worst outcome, with 60% mortality. In the non-infectious group, metabolic disorders and intracranial SOL contributed most to death. The median of ICU-free days was 19.5 (15.0–26.0), showing strong correlation (p<0.001) with both scores (Table 2). Median PICU stay was 7.0 (3.0-14.0) days. Survivors had significantly longer PICU stay compared to non-survivors [median 12.0 (4.0–15.0) versus 5.0 (2.0-9.5) days; p<0.001]. Severe disease courses, prolonged rehabilitation periods, the need for invasive ventilation, and the use of inotropes might be the reasons for such findings. Eighty children received inotropic support, among them 51 needed >2 inotropes at any point in time. Adrenaline was the most frequently used inotrope (76.2%), followed by dobutamine (41.2%), noradrenaline (23.7%), and milrinone (10.0%). VIS ranged from 20 to 70. Eighty-three children required positive pressure ventilation, of which 63 needed endotracheal intubation with a median duration of 4.0 (1.0–7.0) days, and of these, 69.8% died (Table 1). Intubation rate was significantly more frequent in non-survivors (83% versus 17%, p<0.001). 

**Table 2:** Table comparing the mean (±standard deviation) values of Glasgow Coma Scale and Full Outline of Unresponsiveness scores at different time points after pediatric Intensive Care Unit admission in children with altered sensorium:

		Total children (n=100)	Survivors (n=47)	Non-survivors (n=53)	p-value	Correlation with ICU free days	p-values
Glasgow Coma Scale (hours)							
	0	8.40±2.2	9.23±1.4	7.66±2.5	<0.001	0.325	0.001
	12	7.83±2.8	9.68±1.9	6.19±2.5	<0.001	0.608	<0.001
	24	7.75±3.6	10.70±2.3	5.13±2.2	<0.001	0.767	<0.001
Full Outline of Unresponsiveness score (hours)							
	0	9.40±2.9	11.02±1.7	7.96±3.1	<0.001	0.463	<0.001
	12	8.79±3.8	11.47±2.1	6.42±3.4	<0.001	0.608	<0.001
	24	8.30±4.7	12.13±2.6	4.91±3.2	<0.001	0.231	0.231

ICU: Intensive Care Unit.

**Figure 2:**
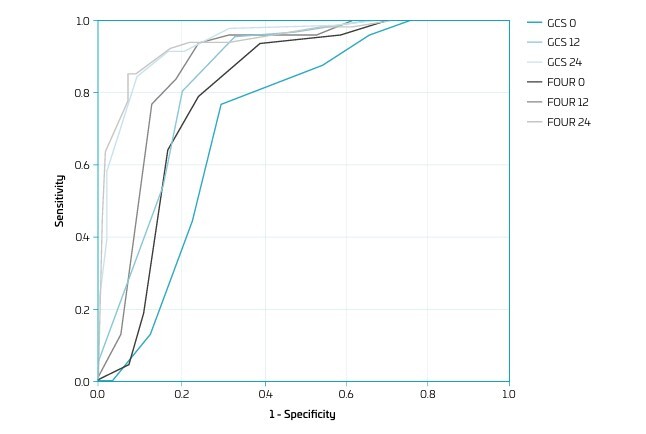
Receiver Operating Characteristics curve, comparing the role of serial Glasgow Coma Scale and FOUR scores in predicting in-hospital mortality of critically ill children with altered sensorium admitted to pediatric Intensive Care Unit.

 Mean GCS scores at admission, 12 hours, and 24 hours were 8.4±2.2, 7.83±2.8, and 7.75±3.6, respectively. Corresponding FOUR scores were 9.40±2.9, 8.79±3.8, and 8.30±4.7, respectively (Table 2). The means of both scores were higher among the survivors with a significant difference (p<0.001). ROC analysis showed good mortality predictive ability of both scores (Figure 2). The AUC-ROC values for subsequent GCS and FOUR scores are listed in Table 3. Observations taken at 24 hours showed the highest AUCs in both cases. GCS-24 had an AUC of 0.941 (95% confidence interval — 95%CI 0.896–0.986), comparable to FOUR-24 (0.927; 95%CI 0.872–0.983). 

**Table 3:** Table showing area under the curve of different time-dependent prognostic parameters to predict mortality using receiver operative characteristics curve and their diagnostic properties.

	AUC	95%CI	p-value	Cut-of	Sensitivity (%)	Specificity (%)	PPV (%)	NPV (%)
GCS 0	0.719	0.616–0.822	<0.001	8.5	76.59	69.81	69.23	77
GCS 12	0.847	0.768–0.925	<0.001	8.5	80.85	79.24	77.55	82.35
GCS 24	0.941	0.896–0.986	<0.001	8.5	85.1	90.56	88.88	87.27
FOUR 0	0.804	0.713–0.895	<0.001	9.5	78.72	75.47	74	80
FOUR 12	0.874	0.799–0.948	<0.001	8.5	93.6	75.47	77.19	93
FOUR 24	0.927	0.872–0.983	<0.001	9.5	85.1	90.56	88.88	87.27

AUC: Area under curve; CI: Confidence interval; PPV: Positive predictive value; NPV: Negative predictive value.

 Variables such as age, weight, inotrope requirement, and duration of PICU stay differed between outcome groups and were included in multivariate logistic analysis. Due to an inadequate data set, intubation duration and dialysis requirements were not included in the model. The subsequent GCS and FOUR scores show high correlations with each other. Therefore, GCS and FOUR scores at a fixed time point were included in each regression model due to the risk of multicollinearity. 

 The multiple regression model demonstrated that GCS-0 [AOR 2.025; 95%CI 1.372–2.990; p<0.001] and GCS-12 [AOR 2.010, 95%CI 1.260–3.206; p<0.001] were independent risk factors for mortality in children with altered sensorium (Table 4). Similarly, all three FOUR scores, FOUR-0 [AOR 0.523; 95%CI 0.373–0.733; p<0.001], FOUR-12 [AOR 0.491; 95%CI 0.328–0.734; p=0.001], and FOUR-24 [AOR 0.505; 95%CI 0.399–0.863; p=0.007] were independent mortality predictors. Each increase in the score at admission, 12, and 24 hours was associated with a 0.477, 0.509, and 0.495-fold decrease in the odds of mortality, respectively. Overall, GCS and FOUR scores showed comparable predictive ability at admission and 24 hours. However, FOUR-24 appeared slightly more reliable than GCS-24 for predicting mortality. 

**Table 4:** A logistic regression model is showing predictive power of the Glasgow Coma Scale and Full Outline of Unresponsiveness scores at different time points in critically ill children admitted to the pediatric Intensive Care Unit.

		Adjusted OR	95%CI	p-value	Hosmer and Lemeshov Test	Nagelkerke R2
MODEL 1						
	GCS 0	2.025	1.372–2.990	<0.001	χ^2^(8)=7.758, p=0.457	0.250
	FOUR 0	0.523	0.373–0.733	<0.001		
Variables included in the model: GCS 0, FOUR 0						
Elimination method: Enter method						
Confounding factors: Age, Weight, PICU duration, Inotrope requirement						
MODEL 2						
	GCS 12	2.010	1.260–3.206	0.003	χ^2^(8)=12.013, p=0.151	0.290
	FOUR 12	0.491	0.328–0.734	0.001		
Variables included in the model: GCS 12, FOUR 12						
Elimination method: Enter method						
Confounding factors: Age, Weight, PICU duration, Inotrope requirement						
MODEL 3						
	GCS 24	2.728	0.932–2.273	0.099	χ^2^(8)=4.780, p=0.781	0.391
	FOUR 24	0.505	0.399–0.863	0.007		
Variables included in the model: GCS 24, FOUR 24						
Elimination method: Enter method						
Confounding factors: Age, Weight, PICU duration, Inotrope requirement						

OR: Odds ratio; CI: Confidence interval; GCS: Glasgow Coma Scale; PICU: Pediatric Intensive Care Unit.

## DISCUSSION

 This study demonstrates the role of the FOUR score as an outcome predictor among non-traumatic critically ill children admitted to the PICU. These results indicate that both pGCS and FOUR score showed comparable prognostic performance at admission and early assessment. However, the FOUR score is a stronger association with in-hospital mortality at 24 hours, suggesting better reliability with serial evaluation. 

 The GCS is widely used in hospital settings and in emergency triage for quick evaluation of consciousness. Although GCS is easy to obtain, its ability is limited in assessing the overall neurological condition. The sub-domains are not equally represented in GCS, and the verbal response is difficult to interpret, particularly in the context of intubation and sedation.^6^ A new 16-point coma scale named “The Full Outline of Unresponsiveness (FOUR) score” was designed by Wijdicks et al. at the Mayo Clinic in 2005 in the neurological-neurosurgical ICU.^14^ It was proven to be a useful tool in adults with stroke^15^ and traumatic^16^ and non-traumatic coma.^17^ The FOUR score is calculated from eye responses, motor response, brainstem reflexes and, respiration, in contrary to three components of the GCS, and the components were given equal weight (0 to 4). FOUR score has certain advantages over the GCS as it provides more neurological details, identifies different stages of herniation, facilitates the detection of locked-in syndrome, does not include verbal response, and thus may have a high prognostic value for intubated patients in the ICU.^18^

 We included children aged one month to 12 years, while the existing reports predominantly enrolled children aged five or older due to the ease of raterevaluation.^9^ Initially, the FOUR score coma scale was validated as a neurological assessment tool in children above two years of age,^7,8^ but later studies showed that it was feasible and reliable even among infants by Czaikowski et al.^11^ Our study is one of the few Indian studies to compare the predictive abilities of FOUR and GCS scores among infants.^19^ It affirms that the FOUR score overcomes the limitations of GCS in assessing neurological status, regardless of age or developmental milestones.^20^ There are discrepancies among the previous studies in this area. Previously, three pediatric studies^5,8,21^ were conducted in non-trauma ICU setups, like our study. Differently, there are similar studies in emergency departments^6,7^ and even in pediatric wards.^9^ The FOUR score has been successfully validated in children with traumatic coma.^7,18,22^ We studied the predictive accuracy of the score in critically ill children in a non-trauma PICU, and our findings were similar to those of other studies on similar populations.^6,9^ Most children under study had infectious diseases (52%), followed by metabolic encephalopathies. Previously, studies from tropical countries had shown a predominance of neuro-infection,^6,9,21^ compared to other studies where brain tumors, and hydrocephalus were the major non-traumatic causes of altered sensorium.^5,8^ It can be concluded that both scores perform well in the pediatric population with tropical neuro-infections.^6^ Moreover, studies of European populations showed a slight superiority of the FOUR score over the GCS in predicting in-hospital mortality.^23^

 The mortality rate in our study was 53%, which is considerably high. Our hospital is a busy tertiary care referral center, serving over 15 million people. A significant portion of the sick children present in late, complicated stages of disease. This fact can be explained by a high pSOFA score during admission [IQR 9.0 (4.0)]. The mean admission pSOFA score in the non-survivor group was significantly higher than that in the survivor group. Additionally, 80% of children needed at least one inotrope infusion during ICU stay, indicating deterioration in their hemodynamic status. Moreover, resource and manpower constraints might contribute to such an outcome. 

 The mean GCS and FOUR values were considerably higher among the survivor group than the deceased one, similar to the observations of other studies.^21^ The ROC curve for in-hospital mortality showed that AUC was the highest in GCS-24, followed by FOUR-24, indicating the fair discrimination power of both scoring systems. The AUCs of both scores in our experience were higher than in most of the existing studies.^14,18,24,25^ Proper rater training and continuous follow-up by a neurologist and an intensivist could have contributed to the higher AUCs. However, in a PICU-based study from Iran,^5^ the authors found that the FOUR score (odds ratio — OR 0.13; 95%CI 0.06–0.29) had a better OR than GCS (OR 2.49; 95%CI 1.44–4.32) for predicting mortality. We calculated the AOR for both scores (2.025 for GCS, 0.523 for FOUR), implying a higher positive predictive value for survival with a rise in score. The AOR was comparable to that reported by Kochar et al.^9^ Overall, most studies agreed that both the pediatric GCS and FOUR score are good predictors of mortality. However, there was no significant difference in the ability to predict mortality. Critically ill children requiring intensive care support are often under the effect of sedative drugs, which potentially affect motor response and eye opening, even though impaired brainstem or respiration reflexes are less likely. On the contrary, all three components of the GCS are affected by sedation.^26,27^ Despite these differences, few studies suggest that the inclusion of brainstem and respiratory components cannot replace the simplicity of GCS in clinical practice.^28^

 Serial assessment played a key role in understanding the association between in-hospital mortality and both scores over time. While both scores were useful at admission, changes in neurological status over the first 24 hours appeared more informative, particularly for the FOUR score. Previously, only one study had analyzed serial readings of scores^21^ and found that GCS at the 0 and 12^th^ hour, and FOUR scores at the 0 and 24^th^ hour were statistically significant predictors of mortality. Notably, while both scores served as effective indicators at admission, the FOUR score demonstrated a stronger association with mortality by the 24^th^ hour in our study. The heterogeneity of the study samples and settings between these studies might contribute to this difference. The longitudinal approach provided insights into the predictive value of the scores and revealed trends in mortality risk over the course of patients’ ICU stays. This supports the concept that dynamic neurological monitoring may provide greater prognostic insight than single time-point measurements in critically ill children. 

 Another study on children with altered level of consciousness (ALOC) found that the FOUR score had 73.53% sensitivity and 87.88% specificity, compared to the GCS (both 79%).^29^ This study divided the whole sample into three cohorts based on the initial FOUR score values. No children were discharged with a FOUR score of 4 or less, yielding a specificity of 100%. Most children with a FOUR score of 9 or higher survived to hospital discharge. Therefore, the study showed higher specificity at lower FOUR scores, meaning that more than 97% of children will die if the FOUR score is 4 or less. Similarly, more than 99% of children might survive with a FOUR score >10, whereas the GCS score has an accuracy of 0.822 (82.2%). The findings were consistent with the findings of our study. 

 The strengths of our study include prospective data collection and serial scoring at predefined intervals. Secondly, this is one of the few studies to observe the predictive accuracy of the FOUR score in the ICU setting among children with non-traumatic alteration of consciousness. Lastly, the confounding factors were adjusted in multivariate analysis. 

 The study’s limitation lies in its design. The single-centered study limited its external validity and did not assess interrater reliability. Additionally, disease-specific predictive performance of the scores could not be evaluated due to the modest sample size. This limitation of internal validity reduced the precision of the cut-off estimates of GCS and FOUR scores. The AUCs of the FOUR score’s components were not examined, thereby failing to demonstrate the predictive efficacy of those components in isolation. Parameters such as PICU-free days, mechanical ventilation-free days, and chronic morbidity could have been considered. However, these morbidity indicators and longer-term neurological outcomes were beyond the scope of the study. 

 In conclusion, both pGCS and FOUR scores are effective tools for outcome prediction in children with non-traumatic altered sensorium admitted to the PICU. While their overall prognostic performance is comparable, the FOUR score demonstrates superior reliability at 24 hours, highlighting its potential value as a serial monitoring tool in critically ill pediatric patients. 

## Data Availability

The database that originated the article is available with the corresponding author.
